# Methodological considerations regarding response bias effect in substance use research: is correlation between the measured variables sufficient?

**DOI:** 10.1186/1747-597X-6-1

**Published:** 2011-01-18

**Authors:** Andrea Petróczi, Tamás Nepusz

**Affiliations:** 1 Kingston University, Faculty of Science, School of Life Sciences, Penrhyn Road, Kingston upon Thames, Surrey, KT1 2EE, UK; 2 The University of Sheffield, Department of Psychology, Western Bank, Sheffield, S10 2TN, UK; 3 Royal Holloway University of London, Department of Computer Science, Egham, Surrey, TW20 0EX, UK

## Abstract

Efforts for drug free sport include developing a better understanding of the behavioural determinants that underline doping with an increased interest in developing anti-doping prevention and intervention programmes. Empirical testing of both is dominated by self-report questionnaires, which is the most widely used method in psychological assessments and sociology polls. Disturbingly, the potential distorting effect of socially desirable responding (SD) is seldom considered in doping research, or dismissed based on weak correlation between some SD measure and the variables of interest. The aim of this report is to draw attention to i) the potential distorting effect of SD and ii) the limitation of using correlation analysis between a SD measure and the individual measures. Models of doping opinion as a potentially contentious issue was tested using structural equation modeling technique (SEM) with and without the SD variable, on a dataset of 278 athletes, assessing the SD effect both at the i) indicator and ii) construct levels, as well as iii) testing SD as an independent variable affecting expressed doping opinion. Participants were categorised by their SD score into high- and low SD groups. Based on low correlation coefficients (<|0.22|) observed in the overall sample, SD effect on the indicator variables could be disregarded. Regression weights between predictors and the outcome variable varied between groups with high and low SD but despite the practically non-existing relationship between SD and predictors (<|0.11|) in the low SD group, both groups showed improved model fit with SD, independently. The results of this study clearly demonstrate the presence of SD effect and the inadequacy of the commonly used pairwise correlation to assess social desirability at model level. In the absence of direct observation of the target behaviour (i.e. doping use), evaluation of the effectiveness of future anti-doping campaign, along with empirical testing of refined doping behavioural models, will likely to continue to rely on self-reported information. Over and above controlling the effect of socially desirable responding in research that makes inferences based on self-reported information on social cognitive and behavioural measures, it is recommended that SD effect is appropriately assessed during data analysis.

## Findings

Prompted by frequent media exposure of high profile doping cases and prevalence reports, the inadequacy of the detection- and sanction-based deterrence to prevent doping has been progressively recognised. Parallel to this development, anti-doping efforts have turned to developing a better understanding of the behavioural determinants that underline the decision to cross the line to the land of prohibited substances. As a result, the number of social science research projects investigating the social aspects of doping has increased, including several papers developing or testing behavioural models and social cognitive processes underlying doping use [1-21]. The comprehensive review commissioned by the World Anti-Doping Agency [22] showed that the overwhelming majority of social science research is based on self-reports with over 100 doping related papers in the social science domain, of which 69 focused on attitudes. Self-report questionnaires comprise over 97% of these studies, in which the potential effect of response bias was seldom mentioned.

Empirical testing of anti-doping interventions is somewhat lagging behind behavioural model work with only a few notable exceptions such as the ATLAS (Athletes Training & Learning to Avoid Steroids) [23] and ATHENA (Athletes Targeting Healthy Exercise and Nutrition Alternatives) [24] for high school athletes. Although empirical evidence has put forward to show the effectiveness of these well known and widely used, school based health promotion and substance abuse prevention programmes, the evaluation in all cases was based on self-reports at both baseline and interval measurement points [25-30].

Self-report is the most commonly employed method in psychological assessment. In addition to the well known benefits of ease of use and information richness, the method has attracted considerable criticism for potentially distorting effects arising from response set and styles [31]. Most of these limitations stem from two fundamental assumptions that the respondent is i) able to self-report and ii) be willing to self-disclose. Hence, the respondent is assumed to have sufficient insight into what is being measured yet no intention to distort his or her responses. Violations of either of these two assumptions can seriously compromise the validity of self-report assessment. Origins of this distortion range from denial through self-deception to deliberate self-impression management, with varying effect on the construct being measured [32]. Self-presentation (socially desirable responding) is one of these potential distortions. Social desirability, a tendency of respondents to reply in a manner that will be viewed favourably by others, is one of the common method variance mechanisms that can create artefactual association. Owing to this social desirability (SD) effect, respondents may deny or deflate their responses about undesirable whilst inflate their answers on desirable attributes and/or behaviour, in particular in situations when the questions drill into socially sensitive issues.

For example, the difficulty in establishing doping prevalence rate via direct self-reports is partly caused by the inconsistent approach to defining doping, setting timeframe and frequency; and partly due to the varying degree of SD effect present in the target populations [33].

On account of the popularity and convenience of self-report methods, in particular when large data set is required for robust statistical analyses, considerable efforts have been made to estimate, and potentially eliminate, the SD effect in research into socially sensitive issues. These endeavours have included ensuring anonymity, using indirect measures and developing tests that are less prone to manipulation. As a last resort, when SD bias is assumed to be present and cannot be eliminated, researchers often include a scale that measures respondents' tendency to give socially desirable answers and correlate these SD scale scores with the target measures.

When socially desirable responding is considered, typically a distinction is made between SD in response set (that is a property of a particular scale) and SD response style, which is an individual difference variable and as such, affects many if not all responses given by the individual [34]. This distinction is important in dealing with SD responding with response set SD being less problematic in psychological assessments as it affects all respondents equally with information not used in absolute levels but compared to other groups' results. However, SD as an individual difference variable could distort the data obtained [35] and may lead to false interpretation if scores were taken at face value. For example, a recent study using objective verification of doping showed that those who falsely claimed abstinence performed on the social cognitive measures as it would be expected from a clean athlete [36].

Although people with certain personality characteristics (i.e. conscientiousness) are known to score high on the SD scales, studies using objective criteria show that in most cases SD scales do not measure individual differences, hence high correlation between the SD scale and other variables indicate significant shared substantive variance [35], thus indicating the presence of SD distortion. A recent review suggests that SD is a motivated process in which respondents deliberately alter the information they report and the extent of this distortion depends on whether the respondent has anything compromising to report and on design features of the survey [37]. Notably, this distortion also presents to a degree when the reporting is done anonymously, i.e. when there is no danger to be embarrassed directly or having consequences of the admitted behaviour.

Despite the fact that methods for testing, controlling and/or managing response bias are available [32], research into doping attitude or other predictors of doping behaviour has seldom considered response bias or made an attempt to i) estimate or ii) partial out variability owing to this effect. The WADA commissioned literature review on the antecedents of doping behaviour concluded that social science doping research would benefit considerably from improvement in research methodology and measurements [22]. In line with this recommendation, this report aims to draw attention to i) the potential distorting effect of SD and ii) the limitation of using correlation analysis between a response bias measure and the individual variables of interest.

**Figure 1:**
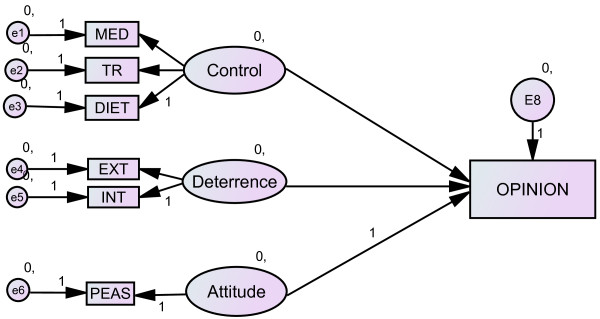
**Model of doping opinion without SD (baseline model). Ovals are constructs (latent variables), rectangles are observed variables; arrows indicate the direction of the relationship**. MED: perceived control over medication, TR: perceived control over training, DIET: perceived control over diet, EXT: self-reported external deterrence factors, INT: self-reported internal deterrence factors, PEAS: explicit doping attitude.

**Figure 2:**
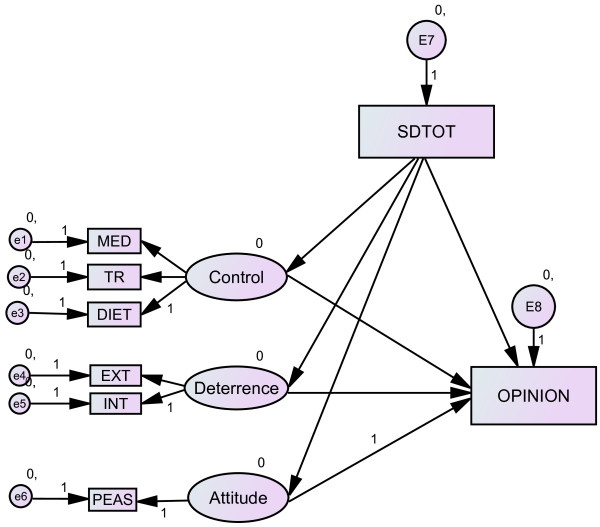
**Model of doping opinion with SD as observed exogenous variable. Ovals are constructs (latent variables), rectangles are observed variables; arrows indicate the direction of the relationship**. MED: perceived control over medication, TR: perceived control over training, DIET: perceived control over diet, EXT: self-reported external deterrence factors, INT: self-reported internal deterrence factors, PEAS: explicit doping attitude, SD: social desirability.

**Figure 3:**
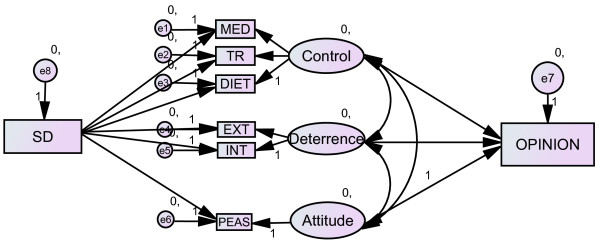
**Model of doping opinion with SD effect on each indicator of the predictor variables. Ovals are constructs (latent variables), rectangles are observed variables; arrows indicate the direction of the relationship**. MED: perceived control over medication, TR: perceived control over training, DIET: perceived control over diet, EXT: self-reported external deterrence factors, INT: self-reported internal deterrence factors, PEAS: explicit doping attitude, SD: social desirability.

**Figure 4:**
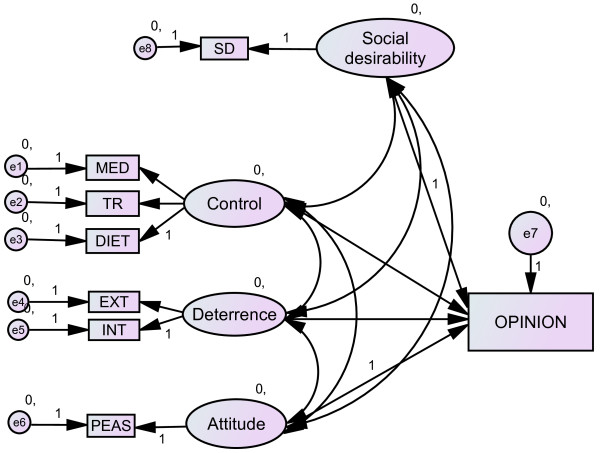
**Model of doping opinion with SD as indicator of the latent variables. Ovals are constructs (latent variables), rectangles are observed variables; arrows indicate the direction of the relationship**. MED: perceived control over medication, TR: perceived control over training, DIET: perceived control over diet, EXT: self-reported external deterrence factors, INT: self-reported internal deterrence factors, PEAS: explicit doping attitude, SD: social desirability.

For this study, a subset of the data used for testing the performance enhancement model [12] was re-analysed using structural equation modeling technique to include a SD variable. The proposed model focuses on opinion formation and is depicted in Figure 1 (baseline model), In line with the current concepts of SD [32,34,35,38] the subsequent models were formulated to test the extent to which the SD is an independent variable that affects the other measured predictor variables at the construct level (Figure 2) and indicator level for the predictor variables (Figure 3); or SD is among the independent predictors affecting expressed doping opinion (Figure 4).

Methods to control SD effects have been widely discussed, with remedies ranging from anonymity to statistical procedures applied [39,40]. At the individual measurement level, SD is either a context specific and temporary effect relating to the response set or consistent across situations related to the person [32,34,35,38]. Although both can affect self-reported responses, it is the latter that may have serious effect on the conclusions drawn from the observed relationship between the measured variables of interest. At the model level, SD is conceptualized as one of the three possible effects: i) suppress genuine relationship, ii) create artefactual relationships or iii) moderate/mediate the relationship between the predictor and the outcome variable [41,42].

Statistical approaches suggest partial correlation and latent variable modeling to test whether SD leads to spurious or suppressor effect [40,41], with a distinction made between suppressor variables and moderator/mediator effects [42]. Notably however, psychometricians speak out against post hoc attempts to statistically partial out SD effect claiming that if doing so, part of the genuine and possibly important variance is also lost [31,38]. Omitting SD when it is a theoretically important variable yields an inadequate model fit [43] and may lead to incorrect conclusions.

Unfortunately, the information on the SD effect on self-reports, particularly in field studies, is limited owing to the difficulty having objective information available on the same person to contrast self-reports on behaviour [35]. Whilst the research has been done on the validity of self-reports on behaviour (i.e. being involved in an act such as drug use, smoking, drinking, etc.), the results are inconclusive. Reassuring validity reports for methods such as the Timeline Follow-Back procedure ([44,45], Drug Abuse Screening Test [46], the CAGE for excessive alcohol consumption [47] or the Cannabis Use Problems Identification Test [48] are counterbalanced by studies using objective verification via biomarkers showing considerable under-reporting of substance use [49-51]. Whilst people may deny their undesirable behaviour for fear of consequences (in case of illegal behaviour), it is equally plausible that such denial is driven by self-presentation. Research showing that SD effect is present even under anonymity [37] supports this notion. Self-presentation plays a particularly important role in research relying on self-reported measures of various psychological constructs such as social cognition and personality. A recent investigation into doping behaviour, benefitting from synergy between social and analytical science, showed that those who denied their compromising behaviour provided answers on the related psychological assessments tapping into attitudes, beliefs and social projection that were congruent with the self-reported (but untrue) behaviour [36].

Therefore the work presented in this paper focuses on the potential SD distorting effect on self-reported measures of various psychological constructs. We used opinion for outcome variable as a construct that results from the combination of someone's beliefs, attitudes, desires, as well as knowledge, understanding and perceptions of a particular situation, including perceived control. Predictor variables were the general doping attitude (Performance Enhancements Attitude Scale (PEAS) [52]), tendency for self impression management (Marlowe-Crowne Social Desirability scale [53], referred to as SD measure in this paper, external and internal deterrence factors and opinion regarding allowing restricted (top level athletes only) and unrestricted (all athletes) use of doping in competitive sport. External deterrence factors were doping control, affordability, perceived use/abstinence of the opponent and disapproval of important others in the athlete's life such as family, friends and coaches. Internal deterrence factors were based on moral values (i.e. doping is cheating, disapproval of drugs) and health concerns.

Correlation coefficients were calculated between SD and other measures. The doping opinion model was tested using structural equation modeling, with and without the self-impression management variable. Scale reliability was assessed using Cronbach's alpha and the KR-21 coefficient. Relationships between the SD and other variables were tested using Pearson and correlation coefficients. Structural equation modeling was performed using AMOS 18 in the PASW package and the R statistical computing software [53] with the SEM package [54]. For further analysis, participants were categorised by their SD score into high- and low SD groups using k-means clustering. All statistical analyses were performed using PASW 18.0.

The data set comprised of 278 college athletes (71.6% male) from Division I & II with the majority from Division II and II/IAA (88.9% combined), mean age 20.1 ± 1.9. Eighty-nine percent of the athletes claimed not having any personal experience with doping, which is congruent with other self-reports but most likely under-reported. Interestingly, 27% of the non-users would support having doping legalized under restricted conditions and a further 3% would even support unrestricted use for high performing athletes. The reliability coefficient values in the present sample ranged from satisfactory to good. Descriptive statistics, along with the scale reliability measures where applicable, are shown in Table 1. Cluster analysis based on SD scores resulted in two distinct groups with a naturally occurring divide at score 17, with cluster centres of 20.39 and 12.18 for high- and low SD groups, respectively. (For details, see Additional file 1: Cluster analysis creating high and low SD groups).

**Table 1:** Descriptive and scale reliability statistics

Variable	Reliability	Mean (SD)	Min - Max in sample	Min - max in scale
Social desirability	0.68	14.93 (4.86)	4 - 31	0 - 33
Doping attitude	0.86	38.24 (12.74)	17 - 83	17 - 102
Control over diet^1^	-	84.49 (18.45)	10 -100	0 - 100
Control over medication taken^1^	-	85.62 (20.68)	0 - 100	0 - 100
Control over training^1^	-	61.21 (25.58)	0 - 100	0 - 100
Internal deterrence	0.68	2.09 (1.49)	0 - 4	0 - 4
External deterrence	0.71	1.56 (1.66)	0 - 6	0 - 6
Legalizing doping for top athletes^2^	-	0.44 (0.6)	0 - 2	0 - 2
Legalizing doping for all athletes^2^	-	0.40 (0.6)	0 - 2	0 - 2

^1 ^Expressed as percentage ranging between 0 (no control at all) and 100 (maximum control)

^2 ^0: absolutely not, 1: with restrictions, 2: without restrictions. Frequencies recorded for legalizing doping for top athletes and all athletes respectively are: 0 = 169 and 180; 1 = 91 and 79; 2 = 15 and 16.

Correlation coefficients between SD and the other measured variables are shown in Table 2. The relationships between social desirability and other doping related measures were in the expected directions. Whilst some were statistically significant, their low value (<|0.22|) suggests that the predictor variables for doping were not strongly affected by socially desirable responding at the measurement level and exhibited an even lower level of correlation (<|0.11|) in the low SD group. However, at the model level, covariances between the latent predictor variables were statistically significant with the covariance between Attitude and Control being considerably larger than the other two (Table 3). Estimated correlations between the predictor latent variables were 0.202, -0.736 and -0.735 for Deterrence - Control, Attitude - Control and Attitude - Deterrence, respectively. Although the model fit could be improved significantly by imposing correlations between the predictor latent variables, we posit that this relationship is largely influenced by the spurious effect of SD. To test this assumption, we tested the models with and without the SD variable where correlation between Deterrence, Control and Attitude were not allowed (Figures 1 and 2). Goodness of fit statistics and fit indices, along with their corresponding customary cut-off values, are summarised in Table 4. Additional file 2 and 3 provides the correlation and covariance matrices (Additional file 2: Pearson r and Additional file 3: Covariance matrix). To facilitate comparison between the models, standardised regression weights and correlations between the latent variables in the doping opinion models are shown in Table 5.

**Table 2:** Strength of relationships between social desirability and predictor variables for the full sample (n = 278) and split samples by high (n = 87) and low (n = 173) SD scores (18 missing data)

	FULL sample		High SD		Low SD	
Predictor variable	Corr. with SD scale (r)	Sign (p)	Corr. with SD scale (r)	Sign (p)	Corr. with SD scale (r)	Sign (p)
Doping attitude	-.219	.001	-.323	.003	.040	.612
Control over diet	.186	.003	.240	.028	.110	.153
Control over medication taken	.073	.245	.041	.707	.032	.681
Control over training	.068	.279	-.092	.400	.074	.337
Internal deterrence	.136	.029	.183	.089	-.006	.939
External deterrence	.047	.448	-.011	.918	.038	.621
Legalizing doping for top athletes	-.184	.003	-.364	.001	-.058	.446
Legalizing doping for all athletes	-.140	.025	-.360	.001	-.047	.543

**Table 3:** Covariances between the latent predictor variables

		Estimate	**S.E**.	z	p
Without SD	Deterrence - Control	2.114	.933	2.265	.024
	Attitude - Control	-73.576	16.495	-4.460	<.001
	Attitude - Deterrence	-3.495	1.093	-3.196	.001
With SD	Deterrence - Control	1.929	.858	2.248	.025
	Attitude - Control	-69.680	16.364	-4.258	<.001
	Attitude - Deterrence	-3.482	1.124	-3.097	.002

S.E.: standard error; z: critical ratio (calculated as estimate/S.E.), p: significance

**Table 4:** Goodness of fit index and comparative fit indices for the doping opinion model depicted in Figure 1

Fit index	*Good fit/Cut-off*	Model without SD	Model with SD
Overall fit	*p > 0.05*	χ^2 ^= 111.0 df = 13, p < .001	χ^2 ^= 39.3, df = 15, p = .001
	*χ*^*2*^*/df <3*	χ^2^/df = 8.544	χ^2^/df = 2.619
CFI	*CFI > 0.9*	.729	0.935
TLI	*TLI > 0.9*	.417	0.845
RMSEA	*RMSEA <0.05*	.165 90%CI = .138, .194	.076 90%CI = .048, .106
PCLOSE	*PCLOSE > 0.05*	<.001	.064

CFI: comparative fit index; TLI: Tucker Lewis coefficient; RMSEA: root mean square error of approximation (<0.8 reasonable, <0.5 is good, > 0.1 is not good); PCLOSE: p of close fit (H_0_: RMSEA = 0.05)

**Table 5:** Standardised regression weights on paths and correlations between the latent variables

Path	Model 1	Model 2	Model 3	Model 4
Control → Opinion	-0.111	-0.212	3.228	2.655
Attitude → Opinion	2.159	0.127	5.363	6.190
Deterrence → Opinion	-0.366	-0.355	2.747	2.551
Social Desirability → Attitude		0.360		
Social Desirability → Deterrence		-0.445		
Social Desirability → Control		-0.459		
Social Desirability → Opinion		0.023		2.612
Social Desirability ← Control				
Social Desirability ← Deterrence				
Social Desirability ← Attitude				
Control ↔ Deterrence		0.171		0.184
Control ↔ Attitude		-0.743		-0.697
Deterrence ↔ Attitude		-0.686		-0.626.
Social Desirability ↔ Control				0.332
Social Desirability ↔ Deterrence				0.170
Social Desirability ↔ Attitude				-0.678

Model 1: without SD, correlation between latent variables not allowed (Figure 1)

Model 2: with SD, correlation between latent variables not allowed (Figure 2)

Model 3: SD effect at the individual indicator measures (Figure 3)

Model 4: SD as an independent predictor variable (Figure 4)

As Table 4 shows, the model without SD variable showed poor fit and had substantial amount of unexplained covariances in the observed data. Including SD dramatically improved the model fit. The overall fit index (chi-square statistics testing H_0_: implied covariance structure is the same as the observed covariance matrix) has changed from very poor fit to a good fit. In an ideal scenario, a good fitting model expected to have non-significant chi-square statistics, but owing to its conservative nature, it is seldom achieved. As an alternative approach, the χ^2^/df ratio is used to assess overall fit where the value for good fitting model is expected to be less than 3. This ratio has dropped from 8.5 to 2.6 when SD was included. All comparative fit indices showed improvement but apart from the Bentler Comparative Fit Index (CFI), they did not quite reach the desired level suggesting that the model can be further improved with imposing further or alternative relationships with the SD variable.

In order to encapsulate the effect SD individually has on the measured predictor variables and whether SD can be considered as an independent predictor for explicitly expressed opinion, we also tested models depicted in Figures 3 and 4, respectively. Both models showed good fit (Table 6). The best model fit was achieved when SD was included as an independent predictor with covariances allowed between the endogenous variables (Figure 4). We also tested this model under high and low SD conditions using data from the high SD group and low SD group independently. Interestingly, both models independently showed good, even improved, fit. This is despite the split sample results showed no significant correlation with the SD (Table 2) and the baseline model without SD (Figure 1) did not show adequate fit (χ^2 ^= 73.40, df = 13, p < 0.001) for the low SD group, The baseline model fit for the high SD group was slightly better but far from being adequate (χ^2 ^= 48.77, df = 13, p < 0.001). Model fit indices for the split sample analysis are shown in Table 7. Descriptive statistics on the measured variables by SD groups are displayed in the Additional file 1: Cluster analysis creating high and low SD groups. This peculiar pattern may be suggesting two things: i) that SD has a model level effect even in cases where SD reported low and ii) SD results in giving deliberate and goal oriented strategic responses to sensitive questions, hence the measures (including the SD) are more congruent within the respective groups than in the pooled data. The latter assumption could be further tested in experimental conditions where the need for giving strategic response is manipulated.

**Table 6:** Goodness of fit index and comparative fit indices for the doping opinion model depicted in Figures 3 and 4

Fit index	*Good fit/Cut-off*	SD effect at the individual indicator measures	SD as an independent predictor variable
Overall fit	*p > 0.05*	χ^2 ^= 25.1 df = 11, p = .009	χ^2 ^= 22.916, df = 13, p = .043
	*χ*^*2*^*/df <3*	χ^2^/df = 2.283	χ^2^/df = 1.763
CFI	*CFI > 0.9*	.962	0.974
TLI	*TLI > 0.9*	.877	0.927
RMSEA	*RMSEA < 0.05*	.0568 90%CI = .033, .104	.052 90%CI = .009, .087
PCLOSE	*PCLOSE > 0.05*	.176	.411

CFI: comparative fit index; TLI: Tucker Lewis coefficient; RMSEA: root mean square error of approximation (<0.8 reasonable, <0.5 is good, > 0.1 is not good); PCLOSE: p of close fit (H_0_: RMSEA = 0.05)

**Table 7:** Model with SD as an independent predictor of the expressed opinion (Figure 4) tested independently with data from the high and low SD groups

Fit index	*Good fit/Cut-off*	High SD group	Low SD group
Overall fit	*p > 0.05*	χ^2 ^= 18.498, df = 13, p = .140	χ^2 ^= 17.793, df = 13, p = .166
	*χ*^*2*^*/df < 3*	χ^2^/df = 1.423	χ^2^/df = 1.369
CFI	*CFI > 0.9*	.951	0.978
TLI	*TLI > 0.9*	.866	0.939
RMSEA	*RMSEA < 0.05*	.070 90%CI = .000, .137	.046 90%CI = .000, .095
PCLOSE	*PCLOSE > 0.05*	.293	.499

CFI: comparative fit index; TLI: Tucker Lewis coefficient; RMSEA: root mean square error of approximation (<0.8 reasonable, <0.5 is good, > 0.1 is not good); PCLOSE: p of close fit (H_0_: RMSEA = 0.05)

The larger than 1 regression weights (Table 5) suggest a suppressor relationship, a statistical phenomenon that often present in social science research using latent variables if collinearity is present in the data [54], affecting the self-reported Attitude measure the most. The high negative correlation between Attitude and SD, which clearly exists and strong in the high SD group (-0.681) but dramatically reduced in the low SD group (-0.293), indicates that SD acts as a suppressor for Attitude measure the most with other indicators are also affected to a lesser degree. Further research is required to determine whether SD effect is a common method variance [39-41] or a theoretically meaningful component [35,43].

In conclusion, despite that the relationship between social desirability and other doping related measures appeared to be reassuringly low, the SEM analysis revealed that the model without the SD variable contained a large amount of unexplained variances resulting in a poor model fit. Including SD increased the proportion of observed covariances explained by the model; improved the fit indices to the desirable level for a satisfactory model fit. Whilst the social desirability bias at the individual variable level was not concerning, the results showed that the accumulated effect at the model level can be quite significant. Large measurement error can result in failing to find robust relationship; hence correlation coefficients may not be able to reflect accurately the effect of socially desirable response in research based on self-report survey data. The presence of social desirability was clearly evidenced when the data were subjected to appropriate statistical tests. This is in line with a recent study showing mediating and moderating effect of social desirability between doping attitudes and susceptibility [57].

Based on the results reported here and in keeping with previous work [36], we propose that conclusions drawn on behavioural models with several determinants of doping (or drug), relying solely on self-reports, should be interpreted cautiously. Repeating some key research with the inclusion and measure of SD effect to provide further evidence for (or falsify) the assumption that SD is a substantial part of the explicit measures of the social cognitive determinants of doping would be a worthwhile endeavour, with a potential to advance the current standing of social science research on doping significantly. In addition to coalescing disparate analytical and social approaches to create a unique platform to investigate sensitive behaviour, progress has also been made in identifying methods that may overcome the limitations associated with the sole use of self-report methodology such as introspective limits and social desirability [37]. In this study, combining self-reported measures with implicit associations in the in the context of objective behavioural information, a distinctive cognitive patterns emerged for those athletes who denied their doping use.

In the absence of direct observation of the behaviour in question (i.e. doping use), evaluation of the effectiveness of future anti-doping campaign, along with behavioural model testing, will likely to continue to rely on self-reported information. Controlling the effect of socially desirable responding is recommended in research that makes inferences based on self-reported information on social cognitive and behavioural measures. Considering SD in study design where it is feasible is strongly recommended [39]. Situations with reduced demand for giving SD responses where respondents are not fully aware of the purpose of the investigation or the options for giving strategically selected responses are not overtly available by the questionnaire design could help reducing SD distortion. For example, implicit social cognition research investigating automatic process underlying social judgements and behaviour has steadily gained popularity in social psychology [58]. The implicit association test (IAT) procedures, relying on latency differences measured on carefully crafted lexical sorting tasks [59-61] are thought to overcome, at least to a degree, the limits associated with and has shown predictive power over and above explicit self-reports for future behaviour [62]. Upon further refinement, a combined explicit and implicit assessment approach can be successfully used in to improve self-report methodology. In cases where SD effect cannot be mitigated via study designs, including statistical analyses to estimate the extent and magnitude of the SD effect in research on the determinants of socially sensitive behaviours is strongly recommended.

Findings from this research should be extended to other variables used for predicting doping. These constructs include but not limited to vulnerability/susceptibility, willingness, motivation and self-efficacy. Owing to the increasing requirement to move from output-based to outcome-based evaluation in drug-prevention, findings and recommendations of this report may be of interest to researchers and practitioners beyond sport and doping.

## Conflicting interest

The authors declare that they have no competing interests.

## Authors' contributions

AP initiated the study and collected the data. AP and TN performed the statistical analyses contributed equally to drafting the manuscript and both approved the final version.

## Supplementary Material

Additional file 1 **Cluster analysis creating high and low SD groups**. Histogram of the SD scores and descriptive statistics of the high- and low SD groups in the data. Click here for file

Additional file 2 **Correlation matrix of the doping opinion model**. correlation matrix (Pearson r) with significance levels. Click here for file

Additional file 3 **Covariance matrix of the doping opinion model**. covariance matrix. Click here for file
