# Supplementary material for: Methodological considerations regarding response bias effect in substance use research: is correlation between the measured variables sufficient?
Source: Subst Abuse Treat Prev Policy. 2011 Jan 18;6:1. doi: 10.1186/1747-597X-6-1 (PMC3037907; doi:10.1186/1747-597X-6-1)
Supplement: Additional file 1 — Cluster analysis creating high and low SD groups. Histogram of the SD scores and descriptive statistics of the high- and low SD groups in the data. [file 1747-597X-6-1-S1.PDF]

Using k-means clustering, 87 were categorised as 'high SD' and 173 were categorised as 'low SD' ($F = 450.411$, $p < .001$)[1] of the total 260 valid cases with 18 missing. The distance between cluster centres was 8.206.

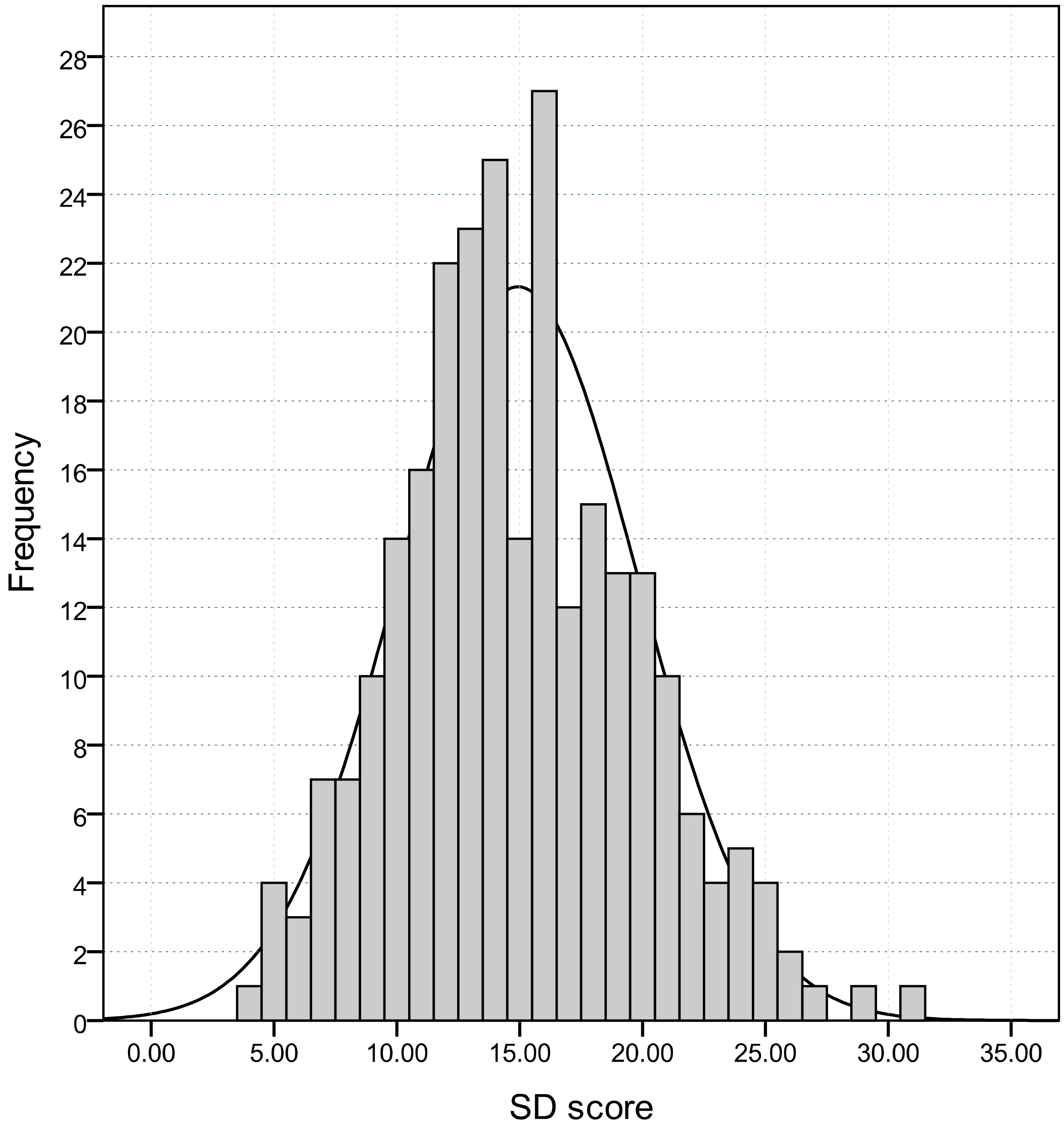

Figure A1: Histogram of the SD scores' distribution in the sample

[1] Note: The F tests should not be interpreted as tests of the hypothesis as the clusters have been chosen to maximize the differences among cases in different clusters.

Table A1: Descriptive statistics of the SD scores the high and low SD groups

	Low SD group	High SD group
Mean score (Standard deviation)	12.185 (2.945)	20.391 (2.935)
Standard error	0.224	0.315
Range (Minimum – Maximum)	12 (4 – 16)	14 (17-31)
Median	13	20
Skewness (standard error)	-0.634 (0.185)	1.173 (0.258)
Kurtosis (standard error)	-0.253 (0.367)	1.440 (0.511)

Table A2: Descriptive statistics of the measured variables in the models by SD groups

	Low SD group		High SD group	
Variable	Mean (SD)	Min – Max in sample	Mean (SD)	Min – Max in sample
Doping attitude	39.83 (12.72)**	19 - 83	35.13 (12.04)**	17.00 - 68
Control over diet[1]	83.44 (20.02)*	10 - 100	88.44 (13.24)*	50 - 100
Control over medication taken[1]	84.92 (20.95)	0 - 100	87.78 (19.97)	0 - 100
Control over training[1]	59.70 (25.89)	0 - 100	63.49 (25.30)	10 - 100
Internal deterrence	1.98 (1.49)*	0 - 4	2.38 (1.47)*	0 - 4
External deterrence	1.62 (1.52)	0 - 4	1.78 (1.97)	0 - 6
Legalizing doping for top athletes[2]	0.48 (0.59)	0 - 2	0.34 (0.59)	0 - 2
Legalizing doping for all athletes[2]	0.42 (0.58)	0 - 2	0.35 (0.61)	0 - 2

[1] Expressed as percentage ranging between 0 (no control at all) and 100 (maximum control)
[2] 0: absolutely not, 1: with restrictions, 2: without restrictions. * Indicates statistically significant difference at $\alpha = 0.05$, ** indicates statistically significant difference at $\alpha = 0.01$.
